# Supplementary material for: Mental health literacy in nursing students: Insights from a cross-sectional analysis
Source: PLoS One. 2025 Jun 5;20(6):e0323728. doi: 10.1371/journal.pone.0323728 (PMC12140200; doi:10.1371/journal.pone.0323728)
Supplement: Attachment — Submitted filename: Response to Reviewers1.docx [file pone.0323728.s003.docx]

**Reply to the Editors and Reviewers:**

Dear Editor:

Thank you for reviewing, and I am addressing your comments.

I have rewritten and changed the manuscript as the reviewers required, addressing their comments below.

|  | **Comments** | **Responses** |
| --- | --- | --- |
|  | **Reviewer #1** |  |
| 1 | Your topic and your manuscript are very good, but in the abstract in method, please explain the analysis method, and in the result, please add the association finding. | Data analysis and association findings were added to the methods section in the abstract, lines: 49-52 & 57-59. |
| 2 | In conclusion, add some sentences for practical using of your finding. | The conclusion section was revised. Study recommendations and implications were added to the conclusion, lines: 311-324. |
|  | **Reviewer #2** |  |
|  | **Abstract** |  |
| 1 | Please correct all parts of the article according to the guidelines of the journal authors guideline. | The abstract format has been modified in accordance with the journal's guidelines. |
| 2 | In the methods section please bring year of performing of this study, sampling methods and data analysis methods | Lines 46-47: The dates of study performance and sampling. Lines 49-52: Data analysis methods. |
| 3 | In the conclusion part, it is necessary to specify the researcher's proposal to improve the conditions and use of the beneficiaries | Lines 59-63: Recommendations of the study to “improve conditions and the use of beneficiaries.” |
|  | **Introduction**  **Please bring the following items:** |  |
| 4 | Definition of the research problem | Lines 93-98: Definition of the research problem. |
| 5 | The magnitude and importance of the study variable | Lines 98-102: Significance of the topic. |
| 6 | Expressing the necessity of conducting the study. | Lines 103-117: Significance of the study. |
| 7 | Finally, the practical purpose of the study should be stated. | Lines 120-122: The practical purpose of the study |
|  | **Methods** |  |
| 8 | Please report the details scoring and validations of study tools | Lines 167-172: Explanation of the tool's validity and reliability measures. |
|  | **Discussion** |  |
| 9 | In the discussion section, it is necessary y to compare the main results of the study with the results of other studies in this field.  To strengthen the article, especially in the introduction and discussion section the following studies are suggested, please used and add to this manuscript references.  Medical tourism development: A systematic review of economic aspects  The effect of bio ethical principles education on ethical attitude of prehospital paramedic personnel  Investigating the burden of disease dimensions (time-dependent, developmental, physical, social and emotional) among family caregivers with COVID-19 patients | Thank you very much for your thoughtful suggestions and for highlighting the importance of strengthening the introduction and discussion sections through relevant literature. We carefully reviewed the suggested studies and recognize their value in their respective areas of research. However, we respectfully note that the focus of our manuscript is specifically on mental health literacy among nursing students, and the recommended references—while insightful—are not directly aligned with the scope or objectives of our study.  To ensure thematic coherence and relevance, we have instead incorporated comparisons with a number of studies closely related to our topic, including: Kristina et al. (2020), Saito and Creedy (2021), Jafari et al. (2021), Dias et al. (2018), Chao et al. (2020), Gorczynski et al. (2020), Aref-Adib et al. (2016), Hjetland et al. (2021), Phalswal et al. (2023), Bizzotto et al. (2023), Chen and Lucock (2022), Gewalt et al. (2022), and Evans-Lacko et al. (2012). These references offer a more direct comparison to our findings and help to contextualize them within the existing literature on mental health literacy.  We sincerely appreciate your input and hope that the revised manuscript meets your expectations. |
|  | **Conclusion** |  |
| 10 | What are your suggestion for future studies? | Recommendations for future studies are included on lines 312-325. |
|  |  |  |
|  | **Reviewer #3** |  |
| 1 | This topic is very crucial. It is important for nursing students to promote mental health awareness and address mental health issues as future healthcare providers. | Thank you for the valuable feedback. |
| 2 | The researchers had a limitation in the sample size " 295" students only | Regarding the study sample size of 295 students, we followed the recommended ratio of 5-10 participants per data collection tool item in our cross-sectional study (Bentler & Chou, 1987; Hair et al., 2019; Green & Young, 1993).  The MHLq scale used in this study consists of 29 items. As such:  Minimum: 145 participants (29 × 5)  Preferred: 290 participants (29 × 10) |
